# Bonded lingual retainer adhesives and discoloration: An in vitro study

**DOI:** 10.1007/s00056-023-00453-7

**Published:** 2023-03-03

**Authors:** Sabahat Yazıcıoğlu, Hasan Karadeniz

**Affiliations:** Faculty of Dentistry, Department of Orthodontics, University of Ondokuz Mayıs, 55139 Atakum, Samsun, Turkey

**Keywords:** Adhesive, Bonded lingual retainer, Discoloration, Liquid polish, In vitro study, Adhäsiv, Gebondeter Lingualretainer, Verfärbung, Flüssige Politur, In-vitro-Studie

## Abstract

**Purpose:**

This in vitro study was conducted to compare the discoloration of a flowable self-adhesive composite, a highly filled composite adhesive, and a liquid polish applied highly filled composite adhesive for bonded lingual retainers.

**Methods:**

Thirty composite discs were fabricated and divided into three groups: group 1, flowable self-adhesive (GC Ortho Connect™ Flow [GCO], GC Orthodontics, Tokyo, Japan); group 2, highly filled composite adhesive (Transbond™ LR [TLR], 3M Unitek, Monrovia, CA, USA); and group 3, highly filled composite adhesive with liquid polish (TLR and BisCover LV™ [TLRB], BISCO Inc, Schaumburg, IL, USA). L*a*b* values were measured by spectrophotometer prior to (T0) and following (T1) immersion in coffee. T1 − T0 differences were calculated as ∆L*, ∆a*, ∆b*, and ∆E*ab values. The Shapiro–Wilk test was performed to determine whether the data were normally distributed. The values that did not fit the normal distribution were evaluated with the Kruskal–Wallis one-way analysis of variance (ANOVA), and Dunn’s test was used for multiple comparisons. The level of significance was *p* < 0.05.

**Results:**

The difference between the TLR and TLRB groups was statistically significant for ∆E*ab (*P* = 0.007). ∆E*ab value of TLR group was greater than ∆E*ab value of TLRB group. The differences between the GCO and TLR groups (*p* = 0.001) and the TLR and TLRB groups (*p* = 0.010) were statistically significant for ∆a*. ∆a* values of GCO and TLRB groups were greater than ∆a* value of TLR group. The difference between the TLR and TLRB groups was statistically significant (*p* = 0.003) for ∆b*. ∆b* value of TLR group was greater than ∆b* value of TLRB group.

**Conclusions:**

Using a Transbond LR polished with BisCover LV or only GC Ortho Connect Flow for lingual retainer bonding reduces coffee-induced discoloration.

## Abstract (de)

**Zielsetzung:**

In dieser In-vitro-Studie wurde die Verfärbung eines fließfähigen selbstadhäsiven Komposits, eines hoch gefüllten Kompositadhäsivs und eines flüssigen, mit Politur aufgetragenen hoch gefüllten Kompositadhäsivs für gebondete Lingualretainer verglichen.

**Methoden:**

30 Kompositscheiben wurden hergestellt und in 3 Gruppen eingeteilt: Gruppe 1, fließfähiges Selbstadhäsiv (GC Ortho Connect™ Flow [GCO], GC Orthodontics, Tokio, Japan); Gruppe 2, hochgefülltes Kompositadhäsiv (Transbond™ LR [TLR], 3M Unitek, Monrovia/CA, USA); und Gruppe 3, hochgefülltes Kompositadhäsiv mit Flüssigpolitur (TLR und BisCover LV™ [TLRB], BISCO Inc, Schaumburg/IL, USA). Die L*a*b*-Werte wurden mit einem Spektralphotometer vor (T0) und nach (T1) dem Eintauchen in Kaffee gemessen. Die Unterschiede zwischen T1 und T0 wurden als ∆L*-, ∆a*-, ∆b*- und ∆E*ab-Werte berechnet. Der Shapiro-Wilk-Test wurde durchgeführt, um zu ermitteln, ob die Daten normalverteilt sind. Die Werte, die nicht in die Normalverteilung fielen, wurden mit der einseitigen Varianzanalyse (ANOVA) nach Kruskal-Wallis ausgewertet, für Mehrfachvergleiche wurde der Dunn-Test verwendet. Das Signifikanzniveau lag bei *p* < 0,05.

**Ergebnisse:**

Der Unterschied zwischen der TLR- und der TLRB-Gruppe war statistisch signifikant für ∆E*ab (*p* = 0,007). Der ∆E*ab-Wert der TLR-Gruppe war größer als der ∆E*ab-Wert der TLRB-Gruppe. Die Unterschiede zwischen der GCO- und der TLR-Gruppe (*p* = 0,001) sowie zwischen der TLR- und der TLRB-Gruppe (*p* = 0,010) waren statistisch signifikant für ∆a*. Die ∆a*-Werte der GCO- und TLRB-Gruppen waren größer als der ∆a*-Wert der TLR-Gruppe. Der Unterschied zwischen der TLR- und der TLRB-Gruppe war für ∆b* statistisch signifikant (*p* = 0,003). Der ∆b*-Wert der TLR-Gruppe war größer als der ∆b*-Wert der TLRB-Gruppe.

**Schlussfolgerungen:**

Die Verwendung eines Transbond LR poliert mit BisCover LV oder nur GC Ortho Connect Flow für das Bonding von lingualen Retainern reduziert durch Kaffee verursachte Verfärbungen.

## Introduction

Bonded lingual retainers may be used long-term or even lifelong by individuals who have undergone orthodontic treatment [1]. Depending on this prolonged use, one of the problems with bonded retainers is discoloration of the bonding adhesive. The type of composite resin material and the polishing procedures used have an effect on the color stability of the composite resin [2, 3]. Chemically cured or light-cured resin composites with different viscosities have been used for bonding retainers [4]. The use of flowable composites has also been suggested for bonding lingual retainers [5]. Reducing the internal and external discoloration of orthodontic adhesives is necessary to reduce the amount of enamel discoloration [6], because the resin tags irreversibly penetrate the enamel structure [7] These resins could discolor because of food dyes or ultraviolet irradiation [6]. Previous studies reported that coffee and tea cause obvious external discoloration of composite resin materials [8]. Polishing with a sealant agent was reported as a method to deal with coffee staining [9].

Liquid polish application, which reduces or eliminates the need for manual polishing [10], can fill voids and reduce surface roughness. It has been shown that composite restorations with a smoother surface tend to be more resistant to staining [2].

Today, coffee consumption has become a regular part of daily life worldwide [11]. Therefore, coffee-induced discoloration of the bonded lingual retainer adhesives can cause enamel color change and difficulty in the diagnosis of caries and plaque. More evidence is needed to define the optimal protocol for lingual retainer bonding [4]. The present in vitro study was conducted to compare the discoloration due to coffee for a highly filled composite adhesive, a liquid polish applied in addition to the highly filled composite adhesive, and a flowable self-adhesive composite used for bonding of orthodontic lingual retainers. The H0 hypothesis of the study was that there is no difference between coffee-induced discolorations of a highly filled composite adhesive, a highly filled composite adhesive combined with a liquid polish, and a flowable self-adhesive composite used in lingual retainer bonding.

## Materials and methods

The study was planned in vitro to eliminate individual differences such as the salivary structure, diet, and oral hygiene habits of individuals. It was designed according to the modified Consolidated Standards of Reporting Trials (CONSORT) checklist [12].

The adhesives Transbond™ LR (3M Unitek, Monrovia, CA, USA) and GC Ortho Connect™ Flow (GC Orthodontics, Tokyo, Japan), which are used in lingual retainer bonding, were tested. BisCover LV™ (BISCO Inc., Schaumburg, IL, USA) was used as liquid polish.

**Fig. 1:**
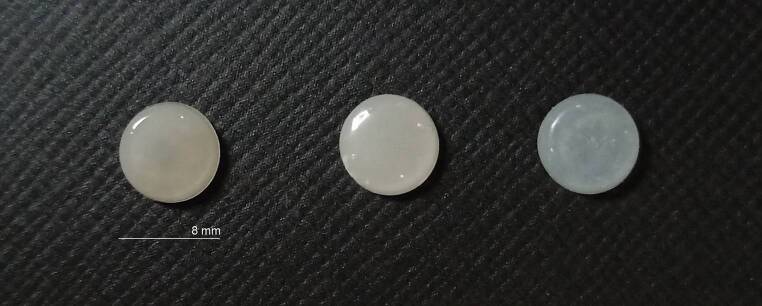
Discs fabricated from lingual retainer adhesives Aus Lingualretainer-Adhäsiven hergestellte Scheiben

According to the ∆E*ab value, which was the primary measurement, the sample size was calculated as 30 specimens in total, with 95% confidence interval (1 − α), 91.2% test power (1 − β), and f = 0.701 effect size [13]. A total of 30 adhesive resin discs (8 mm× 2 mm) were produced (Fig. 1). The groups were as follows: group 1, flowable self-adhesive (GC Ortho Connect Flow [GCO]); group 2, highly filled composite adhesive resin (Transbond LR [TLR]); and group 3, highly filled composite adhesive resin combined with liquid polish (TLR and BisCover LV [TLRB]). After the adhesive resins were placed in a deep transparent drug blister, curing was performed with a light emitting diode (LED) device (Woodpecker LED. B, Zhengzhou Shengxin Medical Instrument Co., Ltd., Zhengzhou, Henan, China) for 20 s for TLR and 10 s for GCO. BisCover LV was applied to all surfaces of 10 specimens in the TLRB group with a bond brush, and after waiting for 15 s, LED light was applied from 0–2 mm distance for 30 s. High-temperature conditions cause increased color changes in composites [14]. Therefore, 30 specimens were kept for 24 h in distilled water at 24 °C room temperature.

The specimens, which were removed from the distilled water and dried, were numbered for blanking by a residency student not involved in the study. Each disc was placed in random order in the covered compartments on which its number was written.

The Commission Internationale de l’Eclairage L*a*b* (CIELAB) values of all specimens were measured by a spectrophotometer (Konica Minolta CM‑5, Osaka, Japan) [15]. The CIELAB color space is approximately uniform and has coordinates for lightness, i.e., white–black (L*); red–green (a*); and yellow–blue (b*) [16]. The D65 standard light source with internal automatic white calibration was used for all measurements and ∆E*ab color differences were calculated.

Before immersion in the discoloring solution the L*a*b* values (T0; Table 1) were measured by one researcher (SY) with 3 repetitions for each disc. The discoloring solution was prepared by mixing 17.5 g of coffee (Nescafe 3 in 1, original, Nestle Türkiye Food, Maslak-İstanbul, Türkiye) with 200 ml of water at 80 °C. This coffee brand was chosen because it is a widely used product in the European market and was available from a normal grocery store. Then, the solution was cooled down to 24 °C room temperature for 3 h and filled into the compartments of the discs. One year of coffee consumption was calculated to correspond to 7 days [17] and so the specimens were kept in the coffee solution for 7 days. The solution was renewed every 24 h. The specimens were removed from the coffee, washed with distilled water, and then dried. Next, the L*a*b* values (T1) were measured again with 3 repetitions for each specimen.

**Table 1:** Mean L*a*b* values prior to immersion in the discoloring solution Durchschnittliche L*a*b*-Werte vor Einbringen in die verfärbende Lösung

T0 Measurements	Groups		
	GCO	TLR	TLRB
L* (mean ± SD)	47.63 ± 2.33	53.47 ± 2.05	46.99 ± 1.53
a* (mean ± SD)	−1.82 ± 0.21	−0.68 ± 0.20	−0.80 ± 0.17
b* (mean ± SD)	−4.0 ± 1.73	−0.10 ± 0.93	−0.65 ± 0.47

*SD* standard deviation, *GCO* GC Ortho Connect Flow, *TRL* Transbond LR, *TLRB* TLR and BisCover LV

For the L*a*b* measurements, T1 − T0 differences were calculated and ∆L*, ∆a*, and ∆b* values were obtained. The ∆E*ab values were calculated using the following formula:

1 $$\Updelta \mathrm{E}^*\mathrm{ab}=[{\left(\Updelta \mathrm{L}^*\right)^{2}}+{\left(\Updelta \mathrm{a}^*\right)^{2}}+{\left(\Updelta \mathrm{b}^*\right)^{2}}]^{1/2}$$

## Statistical analysis

The study data were analyzed with SPSS (version 26, IBM, Armonk, NY, USA). The Shapiro–Wilk test was performed to determine whether the ∆E*ab, ∆L*, ∆a*, and ∆b* values conformed to normal distribution. The values that did not fit normal distribution were evaluated using the Kruskal–Wallis test, with Dunn’s test being used for multiple comparisons. The results were presented as mean and standard deviation and the level of significance was set at *p* < 0.05.

## Results

According to the Kruskal–Wallis test, the null hypothesis of this study was rejected (*p* = 0.007; Table 2).

**Table 2:** Kruskal–Wallis test results Kruskal-Wallis-Testergebnisse

Null hypothesis of this study	Test	Sig	Decision
Distribution of ∆E is the same across GCO, TLR, and TLRB groups	Independent samples Kruskal–Wallis test	0.007**	Reject the null hypothesis

*GCO* GC Ortho Connect Flow, *TRL* Transbond LR, *TLRB* TLR and BisCover LV

**Statistically significant difference (*P* < 0.01)

According to the one-way analysis of variance (ANOVA), the ∆E*ab (*p* = 0.020), ∆a* (*p* = 0.001), and ∆b* (*P* = 0.009) values differed significantly between the groups (Table 3). Multiple comparisons of groups were conducted using a post hoc test, i.e., the Dunn’s test (Table 4). The difference between the TLR and TLRB groups was significant for ∆E*ab (*p* = 0.007). ∆E*ab value of TLR group was greater than ∆E*ab value of TLRB group. The difference between the GCO and TLR groups (*p* = 0.001) and the difference between the TLR and TLRB groups (*p* = 0.010) for ∆a* were significant. ∆a* value of GCO group was greater than ∆a* value of TLR group and ∆a* value of TLRB group was greater than ∆a* value of TLR group. For ∆b*, the difference between the TLR and TLRB groups was significant (*p* = 0.003). ∆b* value of TLR group was greater than ∆b* value of TLRB group. ∆L* values did not differ significantly between the groups. The maximum color change was calculated for the TLR group with an average ∆E*ab value of 3.67. The least color change was calculated for the TLRB group with an average ∆E*ab value of 1.79.

**Table 3:** Between-group one-way analysis of variance (ANOVA) test results Ergebnisse der einseitigen Varianzanalyse (ANOVA) zwischen den Gruppen

Color Parameters	Comparison	Mean Square	F	*p*
∆E*ab	Between groups	9.129	4.538	0.020*
∆L*	Between groups	3.065	2.564	0.096
∆a*	Between groups	0.361	14.844	0.001**
∆b*	Between groups	10.944	5.698	0.009**

*Statistically significant difference (*P* < 0.05), **Statistically significant difference (*P* < 0.01)

**Table 4:** Dunn’s test results for multiple comparison of groups Ergebnisse der Dunnʼs-Tests für multiple Gruppenvergleiche

	Groups			Comparison^a^	*p*
	GCO (a)	TLR (b)	TLRB (c)		
∆E*ab (mean ± SD)	2.42 ± 1.86	3.67 ± 1.39	1.79 ± 0.7	ab	0.283
				ac	0.700
				bc	0.007**
∆L* (mean ± SD)	−0.009 ± 1.44	−0.477 ± 1.15	−1.11 ± 0.39	ab	0.809
				ac	0.113
				bc	0.320
∆a* (mean ± SD)	0.05 ± 0.20	−0.32 ± 0.13	−0.14 ± 0.11	ab	0.001**
				ac	0.059
				bc	0.010**
∆b* (mean ± SD)	2.03 ± 1.81	3.4 ± 1.37	1.35 ± 0.76	ab	0.198
				ac	0.628
				bc	0.003*

*SD* standard deviation,* GCO* GC Ortho Connect Flow, *TRL* Transbond LR, *TLRB* TLR and BisCover LV

^a^*a* GCO group, *b* TLR group, *c* TLRB group

*Statistically significant difference (*P* < 0.05), **Statistically significant difference (*P* < 0.01)

## Discussion

This color change study was carried out for adhesives applied in an area that is not esthetically visible. But, although it is not visible from the outside, for individuals with bonded lingual retainers, any significant color change in this area may cause difficulties in evaluating the adequacy of oral hygiene and the health of the teeth.

In the present study, two adhesive resin materials specially manufactured for lingual retainers were examined in terms of discoloration. TLR is a highly filled light-cured composite adhesive [18], while GCO is a light-cured flowable self-adhesive orthodontic resin, where the primer is integrated into the paste [19].

The coffee that was used in the present study is frequently consumed and is a strong colorant. In addition, it was also used in previous color stability studies [2, 17, 20]. It was reported that coffee caused a great discoloration in all types of composite resins [20, 21] because of adsorption and absorption of colorants [22, 23].

The surface treatments of composite resins were found to have an effect on their coloration [3]. A sealant agent enhances the surface smoothness of the composite [9]. Researchers reported that composites covered by surface sealants demonstrated lower color variation when compared with those to which sealants were not applied [24]. BisCover LV surface sealant was used to form a smooth surface and caused a significant improvement in the surface smoothness of composites [25]. In the present study, only TLR was covered with BisCover LV because GCO does not require polishing or modeling [26].

It has been reported in the literature that control groups stored in distilled water showed visually imperceptible color variation [24, 27]. Therefore, in this study, subgroups treated with immersion in distilled water only were not considered. Because the goal was to evaluate the effect of the colorant and not of the artificial aging processes on the discoloration of retainer adhesives, only coffee staining was used as an artificial aging process.

In the present study, a spectrophotometer, as in previous studies [2, 16, 21, 22], was used to measure color changes. Because colorimetric measurements allow a reproducible method of color determination, they eliminate subjective aspects in visual color comparisons [16]. In the CIELAB color order system, L*, a*, and b* are evenly distributed in a perceptual color space [2]. Therefore, ∆E*ab color difference values have been used very often for dental restorative materials [13]. In the current study, ∆E*ab color differences were also calculated to measure the color changes of the specimens.

The United States Public Health Service defined the limit for acceptable color changes of dental restaurations to be 3.7 units for ∆E*ab. However, Johnston and Kao reported that the tolerances of mismatch determined in vivo are higher than those determined in vitro [28]. In the in vitro study performed by Nasim et al., ∆E*ab values equal to or greater than 3.3 were regarded as clinically perceptible [29]. Douglas et al. reported that the mean acceptability tolerance was 4.0 ∆E*ab units for 95% of the observers in their study [30]. Paravina et al. also classified the ∆E*ab values. According to their classification they defined: ∆E*ab ≤ 1.2 excellent match; 1.2 < ∆E*ab ≤ 2.7 acceptable match; 2.7 < ∆E*ab ≤ 5.4 mismatch: moderately unacceptable; 5.4 < ∆E*ab ≤ 8.1 mismatch: clearly unacceptable; ∆E*ab > 8.1 mismatch: extremely unacceptable [31]. Eliades et al. used a color difference threshold value of 3.7 units for ∆E*ab for orthodontic adhesive resins [7, 23].

In the present study, coffee caused significant discoloration of the tested lingual retainer adhesives. The ∆E*ab value of the TLR specimens was 3.67. This value was found to be clinically unacceptable according to both threshold values (3.3 or 3.7) of ∆E*ab used in previous studies. In contrast to our results, Faltermeier et al. reported that a highly filled adhesive showed greater color stability [6]. The ∆E*ab values for the GCO and TLRB specimens were 2.4 and 1.7, respectively. According to the classification reported by Paravina et al., the ∆E*ab values of GCO and TLRB specimens showed an acceptable match. The clinically acceptable discoloration of the GCO specimens after immersion in coffee is consistent with this composite’s ability to not require any polishing or modeling. In addition, it was found that the color properties of flowable composites are different from those of universal resin composites [32]. In contrast, ElEmbaby et al. reported that the discoloration of the self-adhesive flowable resin composite they tested was clinically unacceptable [21].

Liquid polish application significantly reduced discoloration in the TLRB group compared to the TLR group. This result was consistent with the knowledge that groups covered by surface sealants demonstrated lower color variation when compared with those to which sealants were not applied for composite resins immersed in coffee [24].

The composition of composite resins is related to their discoloration [3]. TLR contains bisphenol A diglycidyl dimethacrylate (bis-GMA) and triethylene glycol dimethacrylate (TEGDMA), whereas GCO contains urethane dimethacrylate (UDMA) and bisphenol A ethoxylate dimethacrylate (bis-EMA) [33]. Materials that do not contain the hydrophilic monomer TEGDMA were more color stable [2]. This explains the greater color change in the TLR group containing TEGDMA compared to the GCO group containing bis-EMA, which is a hydrophobic monomer that increases resistance to staining. The composites containing the hydrophobic resins bis-GMA and bis-EMA showed the lowest discoloration, while hydrophilic materials were stained by hydrophilic colorants [20, 22].

L* represents shade’s brightness [34]. In the present study, the ∆L* value did not differ significantly between the groups. It has been reported that there is a direct relationship between the opacity of a material and its L* value. As the surface roughness of the material increases, so does its opacity, that is, its whitish appearance [23]. This means that the L* value also increases. According to this, the fact that the L* value remained unchanged in the GCO group in our study is compatible with the surface smoothness of this composite. The ∆L* value showed the greatest decrease in the TLRB group. This change, measured as an increase in surface smoothness due to a decrease in opacity, can be explained by the application of the liquid polish.

a* represents the amount of red–green color [34]. In the present study, the differences between the TLR and GCO groups and the TLR and TLRB groups for ∆a* values were significant. At this coordinate, specimens of the GCO group changed to red, while in the TLR group there was a change to green. In the TLRB group, a significantly less green change was measured compared to the TLR group.

As the value of b* becomes more positive, the color becomes yellower and as the value of b* becomes more negative, the color becomes bluer [34]. In our study, the ∆b* values for all groups showed a color change towards yellow. This result is compatible with the knowledge that the yellow colorants of coffee are absorbed into and penetrate the organic phase of the materials [22]. The ∆b* of the TLRB group was the lowest of the three groups and was significantly smaller compared to the TLR group. This result may be explained with the photoactivation of camphorquinone (CQ) in the composition of BisCover LV which was applied to the surface of the specimens in the TLRB group. Usually the color change caused by photoactivation results in a decrease of the yellow chroma and there is a shift toward the blue range [35, 36]. The CQ:amino ratio significantly affects the yellow–blue axis data. There is a direct relationship between b* and ∆E*ab values and the amount of amine [37]. Therefore, in our study, ∆b* and ∆E*ab values showed a significant difference between the TLR and TLRB groups, although being parallel to each other.

The limitation of our in vitro study was that it did not include all factors that may affect the color of the adhesive under clinical conditions. The effects of using flowable self-adhesive GCO or TLR with liquid polish for lingual retainer bonding on periodontal health, caries formation, and lingual retainer bond failure should be the subject of further studies.

## Conclusions

The following results were obtained in our in vitro study:

- Transbond LR was the retainer adhesive with the highest coffee-induced discoloration with a clinically moderately unacceptable ∆E*ab value of 3.67.
- GC Ortho Connect Flow was ranked second for coffee-induced discoloration, with a clinically acceptable ∆E*ab value of 2.42.
- When Transbond LR was polished with BisCover LV a clinically acceptable ∆E*ab value of 1.79 was observed. Thus, it was the retainer adhesive with the least coffee-induced discoloration.
- Using a Transbond LR polished with BisCover LV or only GC Ortho Connect Flow for lingual retainer bonding reduces coffee-induced discoloration.
