# Correction to: Bonded lingual retainer adhesives and discoloration

**DOI:** 10.1007/s00056-023-00499-7

**Published:** 2023-09-21

**Authors:** Sabahat Yazıcıoğlu, Hasan Karadeniz

**Affiliations:** Faculty of Dentistry, Department of Orthodontics, University of Ondokuz Mayıs, 55139 Atakum, Samsun, Turkey

**Erratum to:**

**J Orofac Orthop 2023**

10.1007/s00056-023-00453-7

The article *Bonded lingual retainer adhesives and discoloration*, written by Sabahat Yazıcıoğlu et al., was originally published electronically on the publisher’s internet portal on 03.03.2023 without open access. With the authors’ decision to opt for Open Choice the copyright of the article changed to © The Author(s) 2023 and the article is forthwith distributed under a Creative Commons Attribution 4.0 International License, which permits use, sharing, adaptation, distribution and reproduction in any medium or format, as long as you give appropriate credit to the original author(s) and the source, provide a link to the Creative Commons licence, and indicate if changes were made.

The images or other third party material in this article are included in the article’s Creative Commons licence, unless indicated otherwise in a credit line to the material. If material is not included in the article’s Creative Commons licence and your intended use is not permitted by statutory regulation or exceeds the permitted use, you will need to obtain permission directly from the copyright holder. To view a copy of this licence, visit http://creativecommons.org/licenses/by/4.0/.

